# From past to present: tracing Africa’s path to universal health coverage

**DOI:** 10.3389/fpubh.2025.1540006

**Published:** 2025-03-07

**Authors:** Evaline Chepchirchir Langat, Paul R. Ward, Hailay Gesesew, Lillian Mwanri

**Affiliations:** ^1^Research Centre for Public Health, Equity and Human Flourishing (PHEHF), Torrens University Australia, Adelaide, SA, Australia; ^2^Centre of Excellence in Women and Child Health, Aga Khan University, Nairobi, Kenya; ^3^Department of Health Services, County Government of Kilifi, Kilifi, Kenya; ^4^Tigray Health Research Institute, Mekelle, Tigray, Ethiopia

**Keywords:** health systems reforms, universal health coverage, Africa, health for all, international policies, equitable access

## Abstract

At the 58th World Health Assembly in 2005, the international community charted a course for universal health coverage (UHC), aiming to ensure access to health care for all in need, of sufficient quality, and without causing financial hardship. At the time, barriers to accessing health care were overwhelming, particularly in low-and middle-income countries like Africa. Currently, 50 of Africa’s 54 countries are in various phases of UHC implementation. Some are developing national UHC agendas, while others have made significant progress but still face gaps in meeting UHC targets. This mini review comprehensively examines the literature to understand the temporal and contextual patterns of Africa’s pursuit of health for all, leading up to universal health coverage (UHC). We applied narrative synthesis to identify the patterns, themes, and trends in the literature. Our findings indicate that African countries share similar contextual and chronological patterns of health reforms towards healthcare for all, which mostly emphasized the importance of revitalizing primary health care (PHC). As such, with Africa striving for UHC, continued investment in a robust infrastructure for primary healthcare is essential even as countries implement complex health insurance programs as their UHC approach. This is particularly critical given the severe impact of economic crises and debt burdens on primary healthcare infrastructure four decades ago.

## 1 Introduction

In 2015, the international community through the 2030 agenda for Sustainable Development, charted a course for global development with commitments to universal health coverage (UHC) (1). UHC is defined as ensuring access to quality health care for all who are in need, without causing financial hardship for those receiving care (1). The concept of “health for all” was endorsed by African countries and the world in 1978 (2). However, Africa faced significant challenges in achieving this goal due to the economic crisis of the 1980s, which led to growing disparities in public healthcare quality and access (3).

In the twenty-first century, there was a renewed commitment to the health for all agenda. This commitment was witnessed through the UHC resolution at the 58th World Health Assembly in 2005 (4), the Ougadougou Declaration in 2008 (4), and the Astana Declaration in 2018 (5). These progressive goals called for the revival of primary healthcare and the recognition of healthcare access as a human right. While these declarations emphasized the importance of reducing financial, physical, and other barriers to quality health care, global macroeconomic factors continue to significantly impact how healthcare and UHC approaches are organized and implemented at both the continental and individual country levels in Africa.

In this paper, we explore the four evolutionary periods that played a role in how Africa adopted the concept of health for all, including UHC. We applied narrative synthesis to identify the patterns, themes, and trends in the literature Using examples from various countries, we demonstrate how Africa’s health systems responded to these periods and the progress made towards achieving UHC. Our review highlights the shared challenges and successes across the continent, emphasizing the ongoing need for investment in a robust primary healthcare infrastructure to ensure equitable access to quality healthcare for all citizens.

## 2 The evolutionary periods that shaped healthcare systems in Africa

### 2.1 The 1978 Alma-Ata Declaration—health for all through a primary health care approach

Following the Second World War, the Declaration of Health for All in Alma-Ata in 1978 was launched (6). Alma-Ata was considered a socialist approach to healthcare, as it aimed for the socialization of healthcare with a primary healthcare (PHC) focus (6). PHC was then defined as “essential healthcare based on practical, scientifically sound and socially acceptable methods and technology made universally accessible to individuals and families in the community through their full participation and at a cost that the community and country can afford to maintain at every stage of their development in the spirit of self-reliance and self-determination” (7). Although the health for all approach was noble and sound, it faced criticism from the World Bank and International Monetary Fund (IMF) for being overly ambitious and expensive (8). As a result, several alternative strategies for achieving health were proposed and adopted. One such strategy was selective primary healthcare, which focused on a smaller range of high-impact activities, such as promoting childhood growth, breastfeeding, and immunization (8).

Focusing on PHC is arguably one of the cost-effective ways that some countries have carved their path to increasing service coverage and access to care for its population (9–13). Provision of PHC involves improving the supply of health services through the construction of primary health care facilities, recruiting more staff to run the facilities, ensuring access to medicines and technologies to diagnose and treat medical conditions, and establishing training programs for the human resources serving the primary health care facilities. However, despite the broad vision of UHC, countries in Africa have often focused on the establishment of state-funded insurance as a dominant approach to UHC (14, 15).

Seychelles is an example of an African country that has invested significantly in primary health care. Seychelles adopted the PHC strategy advocated by the WHO a few years after gaining independence in 1976 (16). By the 2000s, two decades after implementing PHC, Seychelles experienced notable improvements in healthcare, including increased service coverage, and significant decreases in infant, child, and maternal mortality rates, as well as better control of communicable diseases (16). These achievements were largely attributed to the government’s unwavering political will, strong citizen advocacy, universal free access to primary healthcare, and substantial government funding, which saw a 65% increase from 2009 to 2015. Additionally, Seychelles benefited from a highly literate population (92%) and a significant healthcare workforce. The country’s consistent investments in the health sector further enhanced service accessibility. Beyond improved health outcomes, the Seychelles’ economy flourished, leading to higher living standards, a benefit the nation continues to enjoy (16).

### 2.2 Introduction of user fees for health under the structural adjustment programs

International development began as a conscious goal in the 1950s, following the Second World War (17). High-income nations provided monetary and technical aid through United Nations (UN) organizations to promote economic growth, which was expected to benefit the general public. The World Bank and IMF were established to monitor this process, together with the UN, which identified the first Development Decade (1961–1970) (17). In the 1980s, the World Bank and IMF became increasingly involved in health system reforms in low-and middle-income countries (LMICs). This marked a shift in focus from investments for economic growth to investments in basic health, nutrition, and education (18).

Due to LMICs’ ballooning debts related to rising interest rates and the global recession of the 1980s, many countries needed loans from the World Bank and IMF to run their economies. Countries receiving these loans were required to comply with the World Bank and IMF’s Structural Adjustment Programs (SAPs), which imposed neoliberal austerity measures. Among other components, SAPs included the introduction of user fees, cutting taxes on upper-income brackets, slashing social spending, privatization of public institutions, deregulation of business sectors, and reduction of public spending on government services (3, 19–21). Within the same decade, the BAMAKO Initiative was launched in 1987 (22), introducing user fees for primary healthcare as a financing approach aimed at increasing the availability of essential drugs and healthcare services in sub-Saharan Africa. User fees for health has however been shown to increase inequity in accessing care in Africa (23), with the poor being pushed into catastrophic and impoverishing states when accessing care (24). Egypt and Tunisia to the north, Ghana and Nigeria to the west, and Tanzania to the east are examples of countries affected by SAPs.

By the late 1980s, Egypt’s health-care system had severe budgetary shortfalls, leading the government to seek aid from international financing bodies (19). These bodies advocated for investing in basic needs such as health, nutrition, and education, while also encouraging privatization and reducing the role of the public sector. Simultaneously, there was a push to improve macroeconomic stability and integration with the global economy through public spending redirection, tax reforms, interest rate adjustments, competitive exchange rates, financial and trade liberalization, and the imposition of user fees (3, 19, 21). During the same time period, Tunisia saw a progressive withdrawal of state funding for social welfare and a diminution in the government’s role in social welfare programs (18). This resulted in lower government health spending and higher out-of-pocket costs, which lowered the quality of health care. Privatization and an injection of foreign direct investment were also observed. Poor quality public health services and health disparities also increased (18).

In Ghana, SAPs had detrimental consequences on access to healthcare, especially for the poor (21). The introduction of user fees put enormous financial pressure on the poor and served as a major barrier to healthcare access, leading to widespread healthcare inequalities. Tanzania, which had aimed to provide free health services through taxation and banned private for-profit medical practice in the late 1970s, suffered greatly following the economic crisis of the 1980s (25). Through SAPs, the country was forced to lift the ban on private for-profit providers and introduce user fees in public health facilities, affecting access to healthcare services for the vulnerable population. In Nigeria, the 1980s economic crisis, coupled with falling oil prices and dwindling public resources, negatively impacted healthcare services (26). The federal government could no longer afford to provide free healthcare and opted to introduce user fees to complement other sources of healthcare funding for all Nigerians.

While the SAP impacted various African countries in the 1980s, its implementation differed across the region based on the country’s debt burden at the time. For example, Seychelles did not experience an increasing debt crisis until 2008, when its foreign debt default made repayment unsustainable, leading the country to seek a bailout from the International Monetary Fund (IMF). Despite taking place in a different decade, the bailout entailed a SAP that also introduced severe cuts in public expenditure, privatization of public services to parastatal companies, and the liberalization of foreign trade, including the removal of restrictions on foreign investment in land and other property (27).

### 2.3 The 58th session of the world health assembly resolution to move towards universal health coverage

Universal health coverage (UHC) became a global priority following the resolution of the World Health Organization (WHO) member states in the 58th session of the World Health Assembly in May 2005 (28). The WHO has provided three dimensions for countries to focus on as they progress towards UHC (24): (i) service coverage, which involves expanding priority services and defining which services to expand first and why; (ii) population coverage, which includes more people and describes who to include first and why; and (iii) financial coverage, which aims to reduce out-of-pocket payments by shifting towards prepayment with clear strategies and rationale.

The UHC resolution also urged member states to restructure their financing to make prepayment the dominant method of financial contribution, thereby discouraging the use of user fees for health (28). In Africa, social health protection schemes through community-based insurance schemes, National Health Insurance (NHI), and social health insurance (SHI) have since dominated as prepayment reforms towards UHC (15, 29). This approach contrasts with the initial approach of “health for all” through primary health care (PHC), which recognizes health as a multisectoral issue and emphasizes community participation, local resources, and trained health workers to tackle health and its social determinants (7).

Nigeria, Gabon, Morocco, Tanzania, and South Africa are examples of countries that have taken up health insurance or are attempting to roll it out as a mechanism for moving towards UHC. In Nigeria, the National Health Insurance Scheme (NHIS) was established in 1999 under Act 35 of the Constitution, and became operational in 2005 as the main vehicle for Universal Health Coverage (UHC) in the country. By 2022, the NHIS had achieved a population coverage of only 39% (30). Additionally, the scheme had accredited just 31% of health facilities, leading to access issues even for insured individuals. The operationalization of the NHIS faces several challenges, including inadequate infrastructure for data monitoring, insufficiently trained staff in health insurance management, limited financial resources, and fragmentation of benefit packages. These factors continue to hinder the effective implementation of the NHIS. In response to the numerous challenges facing the Nigeria’s UHC, in 2022, Nigeria passed the National Health Insurance Authority Act. The act aims to promote, regulate, and integrate Health Insurance Schemes, enhance private sector participation in healthcare service provision, mandate health insurance for all residents of Nigeria regardless of their employment status, and enforce a minimum package of health services that meets national health regulatory standards across all health insurance schemes (31).

In Morocco, UHC was designed using three health insurance schemes introduced in phases: a compulsory health insurance scheme for formal employees, the RAMED scheme for the poor and vulnerable, and a scheme for the self-employed (32). Despite these schemes, collective health financing is limited, with households covering more than half of out-of-pocket health expenditures. The design of the schemes has also led to fragmentation of resource pooling and increased administrative costs (33). Tanzania initially adopted a free healthcare policy but introduced fees for services in health facilities through the structural adjustment program. The National Health Insurance Fund was established in 1999 (34) by the Act of Parliament No. 8 of 1999, and the Community Health Funds act was introduced in 2001 (35). Despite these efforts, health insurance contributes only a small percentage of total health expenditure, with out-of-pocket fees and donor support taking a larger share. In 2022, the government proposed mandatory health insurance, but the bill was withdrawn from parliament twice (25).

In Gabon, UHC began in 2008 with an insurance coverage scheme funded by government tax levies on mobile phone companies and money-sending services, targeting the entire population (36). By 2011, Gabon was close to reaching its entire population, but the scheme is not comprehensive, requiring co-payments for most services. Challenges include false billing by service providers and slow uptake of the insurance by the poor (36). In South Africa, discussions on health care financing have been ongoing since the 1944 Gluckman Commission proposed a fully tax-funded National Health Service to provide free healthcare at the point of service through establishment of primary health care centers (37). Despite numerous policy recommendations from 1994 to 2002 (37, 38), the National Assembly only approved a landmark bill in June 2023 to pave the way for a National Health Insurance (NHI) (39).

Although health insurance is described to be gaining popularity in Africa as the UHC reform of choice, the insufficient supply of health professionals in African countries, the stock-outs of medicines and healthcare equipment, the late payment of rebate payments to health professionals, chronic funding shortfalls to sustain prepayment options, systematic corruption, and implicit rationing in the health system have frequently led to poor or suboptimal outcomes of this approach (12–15, 25, 26, 28–36). Table 1 summarizes the current progress towards UHC made by the African countries mentioned in period 2.1 and 2.2 of this review.

**Table 1:** Current progress towards UHC by country.

Country	The “evolutionary phases”	UHC approach	Country UHC status and progress
Seychelles	The 1978 Alma-Ata Declaration—Health for All through a Primary Health Care Approach	Primary health care approach (PHC)	Seychelles continues to excel in its UHC journey through a PHC approach. The key milestones reflect this progress are, establishing free access to primary healthcare for all citizens, government funded health care with wide service accessibility and a significant health sector workforce of doctors, making consistent health sector investments, with a notable rise in government funding for health (65% increase from 2009 to 2015), benefiting from a highly literate population (92%) and a strong political commitment to healthcare (15, 16).
Egypt	Introduction of user fees for health under the Structural Adjustment Programs (SAPs)	Social Health Insurance (SHI)	By the mid-2000s, health insurance coverage in Egypt was 58%, with out-of-pocket (OOP) expenses at a high of 72%. Health insurance reform attempts between 2005 and 2015 persistently failed due to disagreements between key stakeholders over the goals, proposals, and political processes for change. In December 2017, the Egyptian parliament passed a bill mandating universal health insurance for all citizens, aiming to increase coverage from the current 58% of the insured population (59).
Tunisia	Introduction of user fees for health under the Structural Adjustment Programs (SAPs)	Primary health care approach (PHC) and National Health Insurance (NHI)	Tunisia introduced a social protection system in 1960, featuring health insurance and subsidized or free care. In 1982, the country expanded its healthcare access by implementing a large network of primary healthcare centers. Currently, healthcare delivery is managed by an extensive public healthcare facilities network and a growing private sector. Nearly 90% of Tunisia’s citizens have health insurance, ensuring a good level of basic services. In 2006, Tunisia launched the National Health Insurance Scheme (NHIS) reform, making health insurance mandatory and extended the package of care (50).
Ghana	Introduction of user fees for health under the Structural Adjustment Programs (SAPs)	National Health Insurance Scheme (NHIS)	Ghana established the National Health Insurance Scheme (NHIS) in 2003 through an act of government. The NHIS is primarily funded by tax revenue and offers a uniform benefit package to all members, regardless of their contribution levels or sector affiliation (formal or informal). Enrollment in the scheme is mandatory. Within a decade of its implementation, the NHIS achieved 40% population coverage. However, progress has been slow due to shortages of equipment and medical supplies, leading to restricted access to health services, dissatisfaction, and disinterest among the population in joining the scheme (60).

### 2.4 Refocusing on primary health care for UHC in Africa: the 2008 Ougadougou Declaration and the 2018 Astana Declaration

In 2008, African countries signed the Ougadougou Declaration, revitalizing primary health care (PHC) (4). The goal of the declaration was to re-entrench the principles of achieving health for all, as committed at the 1978 conference in Alma-Ata. Ten years after the Ougadougou Declaration, the 2018 Astana Declaration emphasized the continued relevance and importance of primary healthcare for universal health coverage (UHC) in Africa (40). The Astana Declaration reaffirmed the need for PHC efforts to ensure that everyone enjoys the highest attainable standard of health, regardless of location.

With evidence highlighting the difficulties of providing health insurance without equitable access to good-quality health care services (12–15, 25, 26, 28–36), African countries are now organizing their health systems around primary healthcare principles. These principles include not only a focus on the primary levels of care but also an entirely new conception of health as a key objective across government policies. This includes addressing the social determinants of health, as challenges outside the health sector inherently influence the health of the people and are arguably more expensive to manage. Examples of economies that have focused on strengthening primary healthcare systems as vehicles for UHC include Mali in the west, South Sudan to the east, and Zambia to the south.

In Zambia, following the signing of the Ougadougou Declaration, the Ministry of Health focused on strengthening PHC as a UHC approach, which was formalized in 2008 (41). With a new government coming to power in 2011, the vision for healthcare through this approach was further strengthened, with urban health facilities offering services free of charge. The new government launched a new health policy aimed at providing quality health services to all Zambians as closely as possible to their families. The government also increased the health budget to 15% in line with the 2001 Abuja Declaration (42). South Sudan gained its independence in 2011. Despite the weakening of their health system due to persistent intercommunal conflicts, South Sudan is making positive progress towards UHC. In 2019, it ratified the Health Sector Strategic Plan (HSSP), which includes a plan to roll out the Boma Health Initiative (43). This initiative aims to provide health care at the community level, increase the training and absorption of the health workforce, and develop a health benefit package. Although progress in implementing the HSSP is slow, it has been described as a positive move towards UHC.

Mali, one of the 25 poorest countries in the world, defined a new UHC approach in 2022 (44). Mali has committed to advancing the UHC agenda through the use of a community-based model that aims to take service delivery closer to the doorstep of community members, particularly those in rural and underserved areas. Healthcare services are offered free of charge for children, pregnant women, and those over 70 years of age. This approach is largely funded through government allocation and donor support, including contributions from the World Bank, GAVI, and global funds. This community-based approach has been successful in reducing child mortality by 95% over a seven-year period in Mali. This approach is particularly pivotal for Mali, as it was the country where the Bamako Initiative, which introduced user fees within the Africa region and beyond, was launched. The government plans to introduce tax-based financing and innovative technologies for providing care (44).

## 3 Reflection and conclusion

Reflecting on the journey of health for all and the ongoing quest of UHC in Africa, a complex tapestry of progress, challenges, and lessons learned becomes apparent. Significant efforts have been made in the region to advance health for all, with particular emphasis on revitalizing PHC. This emphasis began with the 1978 Alma-Ata Declaration and continued with initiatives such as Universal Health coverage, the Ougadougou Declaration in 2008, and the Astana Declaration in 2018. The 1980s economic crisis, however, made it extremely difficult for many African countries to meet their 1978 PHC commitments. As a result, there are now widening gaps in the quality and accessibility of public health care, and international policies now play a greater role in determining the African health agenda.

Current evidence indicates that 1.4% of Africa’s population is pushed into poverty due to OOP payments, which is double the global average (45). On average, less than half (48%) of the population in Africa is able to access essential health care services (46), with varying performance between countries (Kenya-55%, Tanzania 46%, Ghana 45%, Rwanda 57%, Nigeria 44%, Gabon 49%, Zambia 54%, Benin 38%). In terms of catastrophic health expenditure, Africa’s performance is promising and better (8.2% at the 10% threshold annually) than the global average of 8.8% (45). However, this needs to be interpreted carefully, as people may be forgoing health care due to unavailability or unaffordability, hence the lower catastrophic expenditure (47, 48).

Despite certain countries like Zambia increasing their public funding for healthcare to 15% (41) as was set in the Abuja declaration (42), its share in most African countries remains considerably below the target. In Nigeria, for example, it is 4.2% (31), 10.5% in Ghana (49), 6% in Tunisia (50), 3.89% in Senegal (51), and 6% in Swaziland (52). With these poor trends, there has been a recent shift to move away from the Abuja target, to a target of government health spending as a percentage of GDP, recommended to be at least 5% of GDP for countries to progress towards UHC (20). According to a study conducted across 10 countries in Africa (53), Rwanda, Kenya, Nigeria, Tanzania, Ghana, Tunisia, Democratic Republic of Congo, Zambia, Egypt and South Africa, the government spending as a percentage of GDP averaged at 4.75% ranging from 3.2% in Ghana to 8.1% in South Africa depicting a commitment from different African countries to channel resources to health in the whole economy.

Findings from this review highlight the need for ongoing investment in the primary healthcare system even as countries work towards implementing more extensive and complex health insurance reforms. Research shows that a well-funded and resourced public primary healthcare system can provide equitable access to high-quality healthcare and address financial barriers to accessing care (9–13, 24, 54–58). This is through investments in: Strengthening the leadership and governance structure in the health care system, investing in an effective and efficient health information system that will support monitoring of the UHC implementation process, reducing financial barriers to access while allocating funds to promote efficiency and equity, strengthening health service delivery to ensure it is of quality, equitable, and meets the priority needs of its people, providing a sufficient and well-trained health workforce motivated enough to provide services that meet patients’ needs, and ensuring access to medicines and technologies to diagnose and treat medical conditions and prevent disability.

In conclusion, the journey of health care evolution in African countries underscores the significant challenges posed by economic constraints and international financial policies. Despite these challenges, notable progress in UHC has been achieved, although disparities persist across different countries. As African countries continue to pursue UHC, it is crucial to address the historical and ongoing impacts of these policies. By drawing on lessons from past experiences, our findings advocate for sustained investment in primary healthcare infrastructure, alongside comprehensive health system reforms, to ensure equitable access to high-quality healthcare for all citizens.
